# Elevated Heart Rate is Associated with Cardiac Denervation in Patients with Heart Failure: A 123-Iodine-MIBG Myocardial Scintigraphy Study

**DOI:** 10.5935/abc.20160166

**Published:** 2016-11

**Authors:** Aline Sterque Villacorta, Humberto Villacorta, Jenne Serrão de Souza, José Antônio Caldas Teixeira, Maria Clara S. S. S. Muradas, Christiane Rodrigues Alves, Bernardo Campanário Precht, Pilar Porto, Letícia Ubaldo, Cláudio Tinoco Mesquita, Antônio Cláudio Lucas da Nóbrega

**Affiliations:** Universidade Federal Fluminense, Niterói, RJ - Brazil

**Keywords:** Heart Rate, Denervation, Heart Failure, Myocardium / radionuclide imaging, Sympathetic Nervous System

## Abstract

**Background:**

In the Systolic Heart Failure Treatment With the If Inhibitor Ivabradine Trial (SHIFT), heart rate (HR) reduction with ivabradine was associated with improved survival and reduced hospitalizations in patients with heart failure (HF). The mechanisms by which elevated HR increases mortality are not fully understood.

**Objective:**

To assess the relationship of baseline HR with clinical, neurohormonal and cardiac sympathetic activity in patients with chronic HF and elevated HR.

**Method:**

Patients with chronic HF who were in sinus rhythm and had resting HR>70 bpm despite optimal medical treatment were included in a randomized, double-blind study comparing ivabradine versus pyridostigmine. This report refers to the baseline data of 16 initial patients. Baseline HR (before randomization to one of the drugs) was assessed, and patients were classified into two groups, with HR below or above mean values. Cardiac sympathetic activity was assessed by 123-iodine-metaiodobenzylguanidine myocardial scintigraphy.

**Results:**

Mean HR was 83.5±11.5 bpm (range 72 to 104), and seven (43.7%) patients had HR above the mean. These patients had lower 6-min walk distance (292.3±93 vs 465.2±97.1 m, p=0.0029), higher values of N-Terminal-proBNP (median 708.4 vs 76.1, p=0.035) and lower late heart/mediastinum rate, indicating cardiac denervation (1.48±0.12 vs 1.74±0.09, p<0.001).

**Conclusion:**

Elevated resting HR in patients with HF under optimal medical treatment was associated with cardiac denervation, worse functional capacity, and neurohormonal activation.

## Introduction

The treatment of heart failure (HF) has improved substantially with the introduction of angiotensin-converting enzyme inhibitors, mineralocorticoid receptor antagonists and beta blockers.^1-3^ Among many effects related to betablockers, the reduction in heart rate (HR) has been recognized for a long time. Many patients with HF are under beta blockers, and some of them remain with HR above 70 bpm despite maximum doses of these medications. Since resting HR in HF is related to increased cardiovascular risk,^4^ it is clinically relevant to search for alternatives to reduce HR.

In the Systolic Heart Failure Treatment With the *I*f Inhibitor Ivabradine Trial (SHIFT),^4^ Ivabradine was compared with placebo in patients with HF and HR above 70 bpm, despite optimal medical treatment. Ivabradine use was associated with improved outcomes, defined as cardiovascular death or hospital admission for HF. The SHIFT Trial confirmed the important role of HR in the pathophysiology of HF.

Increased myocardial sympathetic activity is a prominent feature of HF and is associated with progressive myocardial remodeling, decline in left ventricular function, and worsening symptoms^5,6^. Increased neuronal release of norepinephrine (NE) is usually accompanied by decreased neuronal NE reuptake due to post-transcriptional downregulation of the cardiac NE transporter.^7,8^

The decrease in the NE reuptake mechanism has been successfully assessed by radionuclide imaging with the iodine-123-labeled NE analog metaiodobenzylguanidine (123-I-MIBG). The NE transporter mediates uptake of 123-I-MIBG into myocardial sympathetic nerve endings, and because the compound is not metabolized, the amount of 123-I-MIBG retention over several hours after administration is a reflection of neuronal integrity.^9^ Reduced myocardial 123-I-MIBG uptake has been demonstrated to be an independent predictor of adverse long-term outcome, and improvement in 123-I-MIBG uptake is observed in response to effective HF therapy.^10-12^

Cardiac sympathetic activity is strongly related to HR. Since the mechanisms by which elevated HR increases mortality are not fully understood, we sought to assess the relationship of baseline resting HR with clinical, neuro-hormonal and cardiac sympathetic activity in patients with chronic HF and elevated HR, despite optimal medical treatment.

## Methods

### Study population

This report is part of a larger study, a randomized clinical trial comparing ivabradine with pyridostigmine. It refers to baseline data (before randomization to one of the drugs) of the 16 initial patients included in the trial. Inclusion criteria were the presence of overt HF, sinus rhythm, ejection fraction <50% as assessed by echocardiography (Sympson method), and resting HR over 70 bpm despite optimal medical treatment, including maximum tolerated doses of betablockers. Exclusion criteria were patients with pacemakers, serum creatinine >3 mg/dL, acute myocarditis, active myocardial ischemia, asthma, glaucoma, urinary obstruction, thyroid dysfunction, and patients expected to be submitted to myocardial revascularization or device implantation in the next 6 months. Resting HR was assessed at bedside, after at least 5-min rest, on two consecutive visits before randomization. The Ethics Committee of our hospital approved the study protocol.

Patients were classified according to baseline HR into two groups: a) group 1, patients with HR below or equal to mean HR in the entire population; and b) group 2, patients with the highest HR, above mean HR. Demographic, clinical, laboratorial, and image data were compared between groups. Drug prescription did not change over the last 3 months. Eligible patients who agreed to participate in the study signed a consent form after receiving verbal and written information about the study.

### Myocardial scintigraphy, biomarkers, and functional capacity assessment

Cardiac sympathetic activity was assessed via 123-I-MIBG myocardial scintigraphy. Early and late myocardial anterior planar images were respectively acquired 30 min and 4 h after the radiotracer infusion. Derived from the scintigraphic planar images, semi-quantitative myocardial 123-I-MIBG uptake and washout reflected functional/structural cardiac innervation and sympathetic tone, respectively. The heart/mediastinum ratio (H/M) was determined from the counts/pixel in a visually drawn heart region of interest divided by the counts/pixel in a 7x7 pixel mediastinum region of interest in the mid-line upper chest positioned to reflect the location with lowest activity (i.e., nonspecific background).

Neuro-hormonal activation was assessed by the measurement of N-terminal pro-B-Type natriuretic peptide (NT-proBNP) (Roche Diagnostics, Inc., Indianapolis, Indiana) in blood samples. Functional capacity was assessed by 6-min walk test.

### Cardiopulmonary exercise testing

The MedGraphics (MGC) VO2000 metabolic analyzer was used (Imbrasport, Porto Alegre, Rio Grande do Sul State, Brazil) together with the Ergo PC Elite 13 system and Centurion 300 treadmill (MicroMed, Brasília, DF, Brazil). The gas analyzer was calibrated before each test by the Autocal system in a ventilated setting, and biological calibration was performed monthly. Maintenance of the equipment was carried out by the equipment representative every three months (CAEL, Rio de Janeiro, RJ, Brazil).

The cardiopulmonary exercise test (CPET) consisted of recording of baseline parameters in the first two minutes, a one-minute warm-up at 1 km/h, followed by the ramp protocol. Data analysis was performed by the ErgoPCElite program for Windows 13W (MicroMed, Brasília, Brazil).

At each minute, hemodynamic and electrocardiographic variables were measured, as well as the stress perception using the Borg scale from 0 to 10. The recovery phase took place with the patient seated. The analysis was conducted by two judges, who evaluated the following criteria: VE/VCO_2_ slope, presence of periodic ventilation and establishment of the ventilatory threshold I, from then on called the anaerobic threshold (AT). For the AT, the equivalent ventilatory curves were considered for VO_2_ and VCO_2_, in addition to the exhaled VO_2_ and VCO_2_ fraction curves. The peak VO_2_ was defined as the highest value obtained up to the final thirty seconds, or during the ten seconds of the immediate recovery phase.

### Total body water assessment

Bioelectrical impedance vector analysis (BIVA) was used to assess total body water. This method utilizes the EFG Renal software (Akern, Pontassieve, Florence, Italy) for estimating the parameters of resistance, reactance, and phase angle. Then, the hydration index (HI) was calculated to estimate total body water. Normal HI range is 72.7% to 74.3%; values above this range indicate congestion and values below this cutoff indicate dehydration.

### Statistical analysis

Data are presented as mean ± SD, except for NT-proBNP, expressed as median and interquartile ranges. Categorical variables were analyzed by the chi-square test. A two-tailed unpaired Student's t-test was performed to identify significant between-group differences in normally distributed variables. Mann-Whitney test was used for non-normal data. Statistical significance was accepted at the 0.05 level. The statistical analyses were performed using MedCalc ^®^ software (version 14.12.0, Ostend, Belgium).

## Results

**Figure 1:**
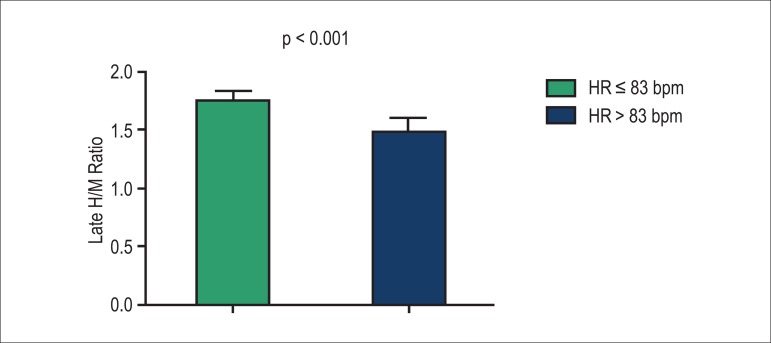
Late heart/mediastinum (H/M) ratio in patients with chronic heart failure according to heart rate (HR) values below or above the population mean

The medications for HF used by the study patients are shown in Table 1. Mean baseline HR was 83±11.5 bpm. Seven (43.7%) patients were in the group with the highest HR (HR above 83 bpm). As shown in Table 2, patients with the highest HR were more likely to be in III/IV New York Heart Association (NYHA) functional class, and had significantly worse functional capacity as assessed by 6-min walk test. There was no difference in systemic congestion between groups as assessed by either limb edema or BIVA. Patients with the highest HR also had higher values of NT-proBNP and lower late (H/M) rate (Figure 1), indicating cardiac denervation. No difference between groups was observed regarding early H/M rate or washout rate.

**Table 1:** Medications for heart failure used by the study population

Medications for heart failure	Results n=16
Carvedilol	16 (100%)
Dose (mg/day)	47.4±7.8
Enalapril	8 (50%)
Dose (mg/day)	34.4±11.3
Captopril	2 (12.5%)
Dose (mg/day)	150±0
Losartan	6 (37.5%)
Dose (mg/day)	100±0
Spironolactone	15 (93.7%)
Dose (mg/day)	25±0
Furosemide	13 (81.2%)
Dose (mg/day)	53.3±24.7
Isosorbide dinitrate and hydralazine	8 (50%)
Dose of hydralazine (mg/day)	157±85.8
Dose of isosorbide (mg/day)	32.6±6.7
Digoxin	5 (31.2%)
Dose (mg/day)	0.18±0.08

**Table 2:** Demographic, clinical, and laboratorial characteristics of heart failure patients separated into two groups according to heart rate values below or above the mean (83 bpm)

Variable	HR≤83 bpm n=9	HR>83 bpm n=7	p value
Age (years)	50.1±13.6	52.4±12.5	0.48
Male gender	4 (44.4%)	5 (71.4%)	0.35
III/IV NYHA functional class	1 (11.1%)	4 (44.4%)	0.10
Limb edema	3 (33.3%)	0 (0%)	0.21
Ischemic cardiomyopathy	2 (22.2%)	1 (14.3%)	0.68
Left bundle branch block	5 (55.6%)	2 (28.6%)	0.35
Minnesota Questionnaire	36.7±18.2	29.7±7.2	0.47
Creatinine (mg/dL)	1.06±0.26	0.97±0.29	0.55
6-minute walk distance (m)	465.2±97.1	292.3±93	0.0029
VO_2_ (mL/min)	17.23±3.62	14.5±5.19	0.58
LV ejection fraction (%)	35.4±15.6	40.5±18.8	0.57
Hydration index (BIVA)	75.8±3.75	73.6±0.05	0.28
NT-proBNP (pg/mL)	378 (140 - 745)	800 (589 - 990)	0.04
Early H/M rate	1.76±0.15	1.69±0.13	0.34
Late H/M rate	1.74±0.09	1.48±0.12	<0.001
Washout rate (%)	37.4±9	34.3±5	0.42

BIVA: bioelectrical impedance vector analysis; LV: left ventricle; NT-proBNP: N-terminal pro-B type natriuretic peptide; NYHA: New York Heart Association; H/M: heart/ mediastinum; VO_2_: peak oxygen consumption.

## Discussion

The main finding of our study was that in patients with chronic HF under optimal medical treatment, elevated HR correlated with neurohormonal activation and cardiac denervation. Cardiac sympathetic abnormalities have been shown to predict outcomes in HF. In the ADMIRE-HF (Adre View Myocardial Imaging for Risk Evaluation in Heart Failure) Study, 961 subjects with NYHA functional class II/III HF and left ventricular ejection fraction ≤35% were included and followed up for up to 2 years.^13^ Event rates were higher for patients with late H/M ratio ≤1.60 as compared with patients above this cutoff (35% vs 15%, p<0.001). Of note, H/M ratio predicted events due to HF progression and arrhythmic events as well. It is important to mention that in the present study, patients with the highest HR had H/M ratio of 1.48, which is within the range shown to predict events in the ADMIRE-HF Study.

Regional cardiac denervation as assessed by positron emission tomography (PET) has also been associated with sudden cardiac arrest. In the PAREPET (Prediction of Arrhythmic Events with Positron Emission Tomography) study, patients in the highest tertile had the highest rates of sudden cardiac arrest.^14^ Thus, either regional or global cardiac denervation indicates an adverse prognosis in patients with HF.^13,14^

In the present study, patients with the highest HR were also the sickest patients as assessed by NYHA functional class, 6-min walk test, and NT-proBNP levels. Of note, such alterations could not be explained by a congestive state. Our study does not permit any speculation regarding a cause-effect relationship between elevated HR and heart denervation. HR could be just a marker of these abnormalities. However, the hypothesis that HR itself is the cause of denervation and high natriuretic peptide levels is supported by the findings of the SHIFT study, in which the reduction of HR with ivabradine, a drug with no neurohormonal activity, led to improvement of outcomes.^4,15^ Besides, the use of ivabradine has been shown to reduce the levels of natriuretic peptides and improve functional capacity.^16^ This hypothesis however needs to be confirmed in further studies.

We found that a simple bedside tool was related to reduced late H/M ratio, reflecting a denervated myocardium. Thus, elevated HR could serve as a screening tool to a more comprehensive evaluation that would include tests such as 123-I-MIBG myocardial scintigraphy in patients with HF. Additionally, HR is a target for treatment in chronic HF and any drug that reduces HR may have a favorable impact on outcomes. A limitation to the present study refers to the small number of patients and the cross-sectional design of the study.

## Conclusion

In summary, we found that in patients with chronic HF and systolic dysfunction, elevated resting HR despite optimal medical treatment was associated with cardiac denervation as assessed by 123-I-MIBG myocardial scintigraphy and neurohormonal activation. These alterations may explain, at least in part, the worse outcomes observed in such patients.
